# Evolution and Role of Proteases in *Campylobacter jejuni* Lifestyle and Pathogenesis

**DOI:** 10.3390/biom13020323

**Published:** 2023-02-08

**Authors:** Bodo Linz, Irshad Sharafutdinov, Nicole Tegtmeyer, Steffen Backert

**Affiliations:** Department of Biology, Division of Microbiology, Friedrich-Alexander-Universität Erlangen-Nürnberg, 91058 Erlangen, Germany

**Keywords:** protease, peptidase, *Campylobacter jejuni*, pathogenesis, lifestyle, virulence factor, anti-bacterial therapy

## Abstract

Infection with the main human food-borne pathogen *Campylobacter jejuni* causes campylobacteriosis that accounts for a substantial percentage of gastrointestinal infections. The disease usually manifests as diarrhea that lasts for up to two weeks. *C. jejuni* possesses an array of peptidases and proteases that are critical for its lifestyle and pathogenesis. These include serine proteases Cj1365c, Cj0511 and HtrA; AAA+ group proteases ClpP, Lon and FtsH; and zinc-dependent protease PqqE, proline aminopeptidase PepP, oligopeptidase PepF and peptidase C26. Here, we review the numerous critical roles of these peptide bond-dissolving enzymes in cellular processes of *C. jejuni* that include protein quality control; protein transport across the inner and outer membranes into the periplasm, cell surface or extracellular space; acquisition of amino acids and biofilm formation and dispersal. In addition, we highlight their role as virulence factors that inflict intestinal tissue damage by promoting cell invasion and mediating cleavage of crucial host cell factors such as epithelial cell junction proteins. Furthermore, we reconstruct the evolution of these proteases in 34 species of the *Campylobacter* genus. Finally, we discuss to what extent *C. jejuni* proteases have initiated the search for inhibitor compounds as prospective novel anti-bacterial therapies.

## 1. Introduction

Campylobacteriosis remains one of the most common zoonotic diseases of bacterial etiology worldwide. With 120,946 confirmed cases, campylobacteriosis was the most reported bacterial zoonosis in the European Union (EU) in 2020, followed by salmonellosis (52,702 cases) and yersiniosis (5668 cases) [1]. The EU numbers may likely be underestimated since the Centers for Disease Control and Prevention (CDC) reported over 60,000 cases in the USA annually, but estimated the actual number of *Campylobacter* infections to be as high as 1.3 million [2]. The main manifestations of campylobacteriosis include acute diarrheal illness often associated with abdominal cramping and fever [3]. In most patients, *Campylobacter* infections last for 1 to 2 weeks with the 24–48 h peak of the illness followed by self-limitation [4,5]. However, if not appropriately treated, the illness may relapse in approximately 20% of infected individuals [3]. In rare cases, post-infectious complications might result in developing serious gastrointestinal disorders including inflammatory bowel disease (IBD) or extra-gastrointestinal diseases such as Guillain-Barré syndrome (GBS), Miller Fisher syndrome (MFS) and reactive arthritis (RA) [6,7].

*C. jejuni* and *C. coli* are the two major *Campylobacter* species that cause campylobacteriosis in the EU with respectively 88.1% and 10.6% of the cases reported in 2020 [1]. Other non-*jejuni Campylobacter* species associated with enteritis account for a minor fraction of disease, including *C. fetus*, *C. upsaliensis*, *C. lari*, *C. rectus*, *C. sputorum* and *C. hyointestinalis*, among others [8]. Campylobacters asymptomatically inhabit diverse animal hosts with the capacity of being transmitted to humans [9]. Major reservoirs of *Campylobacter* spp. that may be transmitted to humans include consumption of contaminated poultry products, water and unpasteurized milk, contact with both domesticated and wild animals, and some other less common sources such as insects and protozoans [10]. The most common causative agent of campylobacteriosis, *C. jejuni*, primarily transmits to humans through inappropriately processed poultry meats, e.g., chicken, ducks and turkeys. Upon infection, *C. jejuni* is preferentially located in the intestinal tract, but has also been isolated from other organs. For instance, Hofreuter and co-authors isolated *C. jejuni* from the liver of mice in a mouse infection model [11]. A follow-up study confirmed that *Campylobacter* spp. could be isolated from the spleen, liver, gallbladder, follicle, upper and lower reproductive tracts and caecum of commercial Leghorn chicken [12].

The capability to colonize various sites of multiple hosts, and thus greatly changing environments, is enabled by a multitude of bacterial virulence and survival factors [13,14,15]. The flagellum is the major bacterial factor that provides effective *C. jejuni* movement, adherence and invasion functions [16]. It consists of the two important subunits, FlaA and FlaB, and can also serve as a type III secretion system (T3SS)-like syringe by secreting or injecting effector molecules such as FlaC, *Campylobacter* invasion antigens (Cia) and flagellar co-expressed determinants (Fed). Several adhesins including Campylobacter adhesion to fibronectin (CadF), fibronectin-like protein A (FlpA), *C. jejuni* lipoprotein A (JlpA) and others provide efficient bacterial attachment to host cells [14]. Various *C. jejuni* strains can release outer membrane vesicles (OMVs) containing cytolethal distending toxin (CDT), which leads to cell cycle arrest and cell death in host epithelia [17]. Finally, a range of proteases facilitates bacterial interaction with host epithelial cells, e.g., by disrupting cellular junctions like the high temperature requirement A (HtrA) protease [18,19,20]. Altogether, they facilitate bacterial survival in changing environments and adhesion to host tissues followed by invasion, replication and spread within the host.

Proteases are of special interest in pathogenesis due to their multiple critical roles in maintaining bacterial sustainability and virulence [21,22,23]. As a basic function, proteases provide cells with nutrients by degrading peptides of various natures [24]. Compared to other enterobacteria, the catabolic versatility of *C. jejuni* is very limited. Its specialized metabolism lacks the common bacterial pathways for processing and utilizing carbohydrates such as glucose and galactose [25,26]. Instead, *C. jejuni* largely depends on the consumption of amino acids, most notably aspartate, glutamate, asparagine, proline and serine, and catabolizes alternative carbohydrates such as lactate and intermediates of the citric acid cycle. Sensing of suitable nutrient sources is mediated by a vast array of chemotaxis proteins, which is followed by nutrient uptake into the bacterial cell. Acquisition of amino acids not only depends on an efficient uptake machinery, but likely also requires digestion of oligopeptides in the immediate surroundings of the bacteria, which is presumably orchestrated by various secreted proteases [25,26]. Further, proteases regulate the bacterial proteome in order to adapt to changing conditions, e.g., under stress pressure [27]. Energy-dependent proteases such as Lon and Clp control the expression of T3SSs in Gram-negative bacteria, mediating injection of pathogenic effector molecules [28]. Secreted HtrA proteases of various Gram-negative bacteria, e.g., *C. jejuni* or *Helicobacter pylori*, can cleave proteins of host cell junctions, which facilitates paracellular migration of the bacteria through the epithelial layer into deeper tissues [29]. Some *Clostridia* exhibit intracellular toxicity to their hosts via metalloproteases in the form of tetanus and botulinum neurotoxins [30]. In this review, we attempt to summarize recent knowledge of the role of *Campylobacter* proteases in pathogen-host interactions. 

Several proteases have already been shown to be involved in *C. jejuni* virulence, and most of them are present in nearly all *Campylobacter* species (Table 1). We start with serine protease HtrA, which is to date the best-studied protease in *C. jejuni* virulence. We then discuss two other serine proteases, Cj1365c and Cj0511, and summarize how the ATPase-dependent (AAA+) proteases FtsH, ClpP, and Lon contribute to *C. jejuni* viability and virulence. Next, we highlight the role of protease PqqE and peptidases PepP, PepF and C26 in *C. jejuni* virulence and discuss the evolution of the proteases within the *Campylobacter* genus. Finally, we lay out possible future research directions.

## 2. Proteases

### 2.1. Serine Proteases

#### 2.1.1. Serine Protease HtrA (Cj1228c)

Members of the family of high temperature requirement A (HtrA) serine proteases are expressed both by prokaryotic and eukaryotic organisms [20,31,32]. The *C. jejuni*-encoded HtrA operates as a bifunctional protein, comprising both protease and chaperone activities, and consists of a signal peptide at the N-terminus, a trypsin-like protease domain and two consecutive PDZ domains at the C-terminus [29]. The HtrA enzyme forms proteolytically active trimers, hexamers and dodecamers in the periplasm [33]. Like many other bacterial HtrAs, a major function of *C. jejuni* HtrA is the protection against a variety of external stress conditions [34,35]. Initially, the importance of HtrA during *C. jejuni* infection was studied in two mouse models, infant wild-type (wt) mice and gnotobiotic interleukin-10 (IL-10)^-/-^ knockout mice [36]. In both animal models, the intestinal colonization loads of *C. jejuni* wt and HtrA mutant strains were similarly high. When infected with wt *C. jejuni*, IL-10^-/-^ deficient mice revealed disturbed crypt structures and strong upregulation of colonic apoptotic cells [37]. This scenario resulted in the infiltration of immune cells such as monocytes, macrophages and neutrophils in the colon, which was associated with elevated release of nitric oxide (NO) and pro-inflammatory cytokines TNF, IFN-γ, IL-6 and MCP-1 [37,38]. Infected IL-10^-/-^ knockout mice also displayed inflammatory reactions in the lung, liver and kidney, demonstrating that immune-relevant reactions were not restricted to the colon. Both phenotypes were dependent on HtrA and its protease activity. *C. jejuni* infection of infant mice revealed comparable results [39]. Taken together, HtrA emerged as a novel *C. jejuni* virulence factor, whose protease activity intensifies campylobacteriosis by causing apoptosis and pro-inflammatory immune pathology in mice [38].

Infection studies using cultured intestinal epithelial cells in vitro have shown that expression of HtrA is required to attach to and enter cells, probably mediated by its chaperone activity on one or more *C. jejuni* adhesins [18,35,40,41,42,43]. In addition, HtrA was shown to be secreted in the extracellular environment, either as a soluble enzyme or as a part of shed OMVs [18,44,45,46]. Remarkably, by using transwell filter systems it was demonstrated that wt *C. jejuni* can effectively and quickly transmigrate across polarized epithelial cells, a process that is associated with cell damage [18,47]. This phenotype is largely abolished during infection with a ∆*htrA* deletion mutant and with a mutant in which the protease function, but not the chaperone function, was inactivated. Those data suggested that HtrA protease activity is required for *C. jejuni* transmigration through the epithelium and led to the proposal that secreted HtrA may disrupt cell junctional proteins between neighboring intestinal epithelial cells. Indeed, *C. jejuni* HtrA was shown to cleave two tight junction proteins, occludin [47] and claudin-8 [19], and the exact cleavage sites were identified. In addition, HtrA cleaves the major protein of the adherens junctions, tumour suppressor E-cadherin, in vitro and during infection in vivo [18,48]. Cleavage of occludin, claudin-8 and E-cadherin results in the disruption of intestinal epithelial cell-to-cell junctions, which compromises epithelial barrier functions and paves the way for *C. jejuni* to travel across the intestinal epithelium using a paracellular pathway [49]. The capability for transepithelial migration can explain why *C. jejuni* expresses two adhesins with high affinity to fibronectin, CadF and FlpA [50]. Fibronectin is the natural ligand for the basal integrin-β1 complex that is important for cell entry of the bacteria [18,50,51,52,53,54]. This way, *C. jejuni* may also be capable of entering the lamina propria, the bloodstream and other organs. By using transwell filter assays, further work showed that *C. jejuni* also facilitated effective translocation of microbiota such as *Lactococcus lactis* or *Escherichia coli* through the epithelial layer. This HtrA-mediated transmigration of microbiota to the basolateral compartment, and thus deeper tissue layers, might be involved in the development of inflammatory bowel disease (IBD) [55].

Because of these important functions and the ubiquitous presence of the *htrA* gene in *C. jejuni*, this protease represents a supreme target for anti-*C. jejuni* therapy. Recent studies in mice and in cell culture systems showed that application of the polyphenolic compound curcumin reduced *C. jejuni*-triggered disruption of the epithelium [56]. Similar results were obtained using vitamin D that diminished *C. jejuni* transepithelial migration [57]. Therefore, vitamin D and curcumin might represent alternative options for the management of *C. jejuni* infections. Finally, HtrA could be a promising target for the development of a vaccine and small inhibitor compounds [58,59,60,61]. However, additional studies are required to improve the specificity of currently available HtrA inhibitors.

#### 2.1.2. Serine Protease Cj1365c

*C. jejuni* protease Cj1365c belongs to the subtilisin-like family of serine proteases. Similar to HtrA, Cj1365c possesses a catalytic triad composed of a histidine, an aspartate and a serine, but the order of the residues in the protease (Asp-Ser-His) differs from that in HtrA (His-Asp-Ser). The domain architecture of Cj1365c consists of an N-terminal S8 peptidase domain and a C-terminal autotransporter domain that provides protease translocation across the membrane, consistent with a predicted location at the outer membrane (Table 1). Intriguingly, proteins containing autotransporter domains are often associated with bacterial virulence [62]. The assumption that Cj1365c may play a role in *C. jejuni* virulence was recently confirmed by a study in an in vitro model of intestinal epithelia. Cj1365c was found in secreted *C. jejuni* OMVs, along with serine proteases HtrA and Cj0511, as well as with approximately 150 other proteins, including important *C. jejuni* virulence factors such as CDT, the adhesins CadF and FlpA and the major antigenic protein Peb3 [44,45]. *C. jejuni* OMVs were shown to possess proteolytic activity that was associated with HtrA, Cj0511 and Cj1365c. Accordingly, proteolytic activity was reduced by deletion of either of the *htrA*, *cj0511*, or *cj1365c* genes [45]. Incubation of cultured T84 cells with OMVs efficiently hydrolyzed the host junctional proteins occludin and E-cadherin, an activity that was associated with HtrA and Cj1365c [45]. As expected from these observations, a knockout mutation of the *cj1365c* gene or pretreatment with serine protease inhibitors resulted in reduced adhesion and invasion of bacteria. In addition to junctional proteins, Cj1365c was proposed to cleave the major endoplasmic reticulum chaperone protein BiP/GRP78 [63], but the biological relevance of BiP/GRP78 protein cleavage in *C. jejuni*-host interactions remains to be elucidated. Interestingly, exposure of *C. jejuni* to physiologically relevant concentrations of sodium taurocholate, a bile salt secreted by the gallbladder into the digestive tract, resulted in increased concentrations of virulence-associated factors within the OMVs and in increased proteolytic activity [63]. 

However, not all *C. jejuni* isolates possess the Cj1365c protease. Our genome-wide screen revealed 2,359 genomes in the RefSeq Genome Database, of which only 1896 contained the *cj1365c* gene. The gene was predominantly found in clinical isolates and isolates from livestock, but not in isolates from environmental sources such as sand of a bathing beach [64]. This gene was prevalent in multiple *C. jejuni* clonal complexes (CCs), which are groups of related strains based on Multi Locus Sequence Typing (MLST). These CCs contained human clinical isolates as well as livestock isolates from cattle, chicken and turkey, indicating no specific host association. In contrast, CC45 and CC283 (mostly human and chicken isolates) and CC1332 (mostly from turkeys, chicken and humans) did not possess *cj1365c* [65]. 

Moreover, our analysis showed that only 60 out of the 1182 *C. coli* genomes in the NCBI RefSeq Genome Database possess the Cj1365c protease. It is missing in the majority of the isolates, including the *C. coli* type strain FDAARGOS_735. In those isolates that carry the gene, the protease shows 97.1% to 99.9% protein similarity to *C. jejuni* Cj1365c, in contrast to between 60% and 80% similarity in other proteins. These observations suggest a recent import of gene *cj1365c* from *C. jejuni* into *C. coli* by lateral DNA transfer. In addition, we searched for Cj1365c among a variety of *Campylobacter* species and found that this protease is absent from the majority of the analyzed species (Table 1). Further studies are required to confirm Cj1365c-mediated cleavage of occludin, E-cadherin and BiP/GRP78 during infection in vivo, and to unravel the biological relevance of BiP/GRP78 cleavage in *C. jejuni*-host interactions.

#### 2.1.3. Serine Protease Cj0511

Another serine protease is Cj0511 [66], which is N-glycosylated in *C. jejuni* [67]. Cj0511 belongs to the family of S41 family peptidases, which are serine endopeptidases similar to the C-terminal-processing protease CtpA from *Bartonella bacilliformis*. Similar to HtrA, C-terminal-processing proteases (CTPs) are implicated in protein folding and processing in the periplasm and contain a PDZ domain that is assumed to mediate substrate recognition [68]. However, while the catalytic triads of both HtrA and Cj1365c are composed of a histidine, an aspartate and a serine residue (HDS-motif), CTPs possess a serine-lysine catalytic dyad (SK-motif). CTPs are critically important for post-translational protein processing, specifically for processing the C-terminus of their protein substrates, hence the name. In addition, CTPs have been associated with regulation of gene expression, stress response, maintenance of cell envelope integrity and bacterial virulence [68]. For example, the *Legionella pneumophila* protein Tsp is implicated in heat stress response and is essential for efficient infection of and intracellular growth in amoeba [69]. CtpA of *Bordetella bronchiseptica* is involved in post-translational processing of filamentous hemagglutinin FHA, a major virulence factor required for adhesion and persistence in the airways of an infected host. Accordingly, a CtpA-deficient mutant failed to persist in the lower respiratory tract [70]. Likewise, deletion mutants of CTPs from *Acinetobacter baumannii* [71], *Brucella suis* [72], *Burkholderia mallei* [73], *Pseudomonas aeruginosa* [74] and *Staphylococcus aureus* [75] exhibited reduced virulence compared to the respective wt bacteria.

Serine protease Cj0511 appears to play a pivotal role in *C. jejuni* biology, as it is present in all published *C. jejuni* genomes. The protease contains a signal peptide spanning 34 amino acid residues. In addition to a predicted cellular location at the cytoplasmic membrane and in the periplasm (Table 1), Cj0511 was suspected to be secreted [76], which is in agreement with the observation that Cj0511 was found in purified *C. jejuni* OMVs [44,63]. Cj0511 appears to play an important role during *C. jejuni* chicken colonization. Cj0511 was highly expressed in an efficient chicken-colonizer strain, but barely expressed in a strain that colonized chickens poorly [76]. Expectedly, a *cj0511* gene deletion mutant was severely impaired in its ability to colonize chickens compared to the parental wt strain. Not only were more chickens of the cohort infected, but the wt strain also infected the chicken ileum and cecum in significantly higher numbers than did the *cj0511* deletion mutant [66,77]. Likewise, deletion of *cj0511* impaired the ability of *C. jejuni* to colonize mice, reducing bacterial numbers by over two logs [78]. The observed differences in colonization efficiency may largely be associated with the reduced ability of the Δ*cj0511* mutant to adhere to and invade IECs.

Exposure of *C. jejuni* to pancreas-secreted amylase triggered the production and secretion of a bacterial α-dextran, a response that depended on a functional Cj0511 [77]. The α-dextran production increased *C. jejuni* stress resistance and facilitated prolonged survival at extra-host temperatures (20 °C and 4 °C), suggesting a crucial role during transmission between hosts, including survival on meat and dairy products prepared for human consumption. In addition, the α-dextran greatly increased *C. jejuni*-induced mortality in the *Galleria mellonella* larvae infection model, an effect that was absent in the *cj0511* deletion mutant, and promoted biofilm formation of *C. jejuni* bacteria in a Cj0511-dependent manner [77]. Yet, the exact mechanisms of how Cj0511 mediates interaction with IECs, how Cj0511 increases virulence during *G. mellonella* infection, how Cj0511 promotes colonization of the chicken ileum and how Cj0511 stimulates biofilm formation remain to be elucidated.

### 2.2. AAA+ Group Proteases

(AAA+) group proteases are widely distributed in all living organisms and are associated with diverse cellular activities. In bacteria, those proteases mostly facilitate survival under various stress conditions [27,79]. The AAA+ (or ATP-dependent) proteases share some distinct structural similarities and specifically contain ATPase (AAA+ module) and peptidase domains. The two major segments are responsible for substrate recognition, unfolding and degradation. Thus, AAA+ proteases regulate proteolysis and control the bacterial proteome in response to cellular needs. During infection, some pathogens strictly depend on the function of such proteases, such as the causative agent of leptospirosis in animals and humans, *Leptospira interrogans* [80]. Inactivation of the *clpB* gene that encodes the AAA+ protease in *L. interrogans* decreased bacterial tolerance to oxidative stress. In addition, gene deletion also hampered bacterial virulence in the gerbil model of acute leptospirosis [81]. Several lines of evidence indicate crucial roles of Clp protease in the virulence, resistance and persistence of *Staphylococcus aureus*, including methicillin-resistant (MRSA) strains [28,82]. As of today, FtsH, Lon and ClpP may be distinguished as AAA+ proteases playing significant roles in *C. jejuni* survival and virulence as discussed below.

#### 2.2.1. ClpP Protease (Cj0192c)

As with most AAA+ proteases, the ClpP protease of *C. jejuni* is involved in heat stress response and protein quality control. Structurally, *C. jejuni* ClpP does not possess the ATPase module and instead forms hetero-oligomeric complexes with the ClpA and ClpX ATPases. In *C. jejuni*, ClpP plays various functions, contributing to both stress tolerance and virulence. Moreover, ClpP was shown to play a major role in the natural competence of *C. jejuni* as the deletion of the *clpA* or *clpX* genes reduced DNA uptake by 5 to 10-fold, respectively, and a Δ*clpP* mutant was virtually untransformable [83]. Knockout mutations of *clpX* and *clpP*, but not *clpA*, showed increased heat sensitivity at 42 °C, suggesting a crucial role of the ClpXP complex in the *C. jejuni* heat shock response [84]. Notably, ClpP was also proposed to promote *C. jejuni* survival at refrigerator temperatures [85]. Thus, similar to the Cj0511 serine protease discussed above, ClpP may increase survival in extra-host environments, and thus play an important role during transmission. While being involved in temperature adaption, ClpP does not seem to be involved in oxidative stress response; aerotolerant and non-aerotolerant *C. jejuni* strains displayed no significant differences in *clpP* gene expression under aerobic conditions [86].

Similar to HtrA, Cj0511, and Cj1365c, ClpP was found to be present in purified OMVs [63], in addition to a predicted cytoplasmic location in the bacterial cell (Table 1). Interestingly, loss of either *clpX*, *clpA* or *clpP* genes impaired bacterial invasion of IECs without affecting *C. jejuni* adherence to the cell surface [84]. Furthermore, the invasion-associated process of *C. jejuni* autoagglutination was dependent on ClpX and ClpP, but not on ClpA. The motility of bacteria was reduced upon deletion of *clpP*, but not *clpA* or *clpX*, which indicates that both ClpAP- and ClpXP-mediated proteolyses may be important in this process. Furthermore, ClpP protein expression was found upregulated in *C. jejuni* biofilm-associated cells. 

Overall, the ClpP protease seems to play a vital role in both maintaining *C. jejuni* viability under heat stress conditions and during invasion of host organisms. We found this protease present in all *C. jejuni* isolates in the NCBI RefSeq Genome Database and that the protease is very conserved within the *Campylobacter* genus (Table 1). Interestingly, Ghunaim and co-authors found the *clpP* gene in only 83.3% of *C. jejuni* isolates [87]. The interstrain variability in virulence gene transcription could probably explain that ClpP was missing in a set of isolates. Thus, the two *C. jejuni* strains NCTC11168 and DFVF1099 displayed different levels of *clpP* transcription after 24 h incubation at 4 °C [88]. In contrast, Wurfel and co-authors found the *clpP* gene present in almost all *C. jejuni* strains isolated from poultry meat products in Brazil [89]. Further studies, including large-scale genome comparisons, are required to clarify whether the Clp protease complex genes are present in all *C. jejuni* strains.

#### 2.2.2. Lon (Cj1073c)

Lon proteases are well characterized in numerous bacteria, including *E. coli* and *S. aureus*. In *C. jejuni*, the Lon protease, which is predicted to be a cytoplasmic enzyme (Table 1), was first identified in cells exposed to heat shock at 48 °C [90]. Dot blot assays revealed a rapid increase in the *lon* mRNA level already after 5 min incubation of *C. jejuni* at 48 °C, reaching the maximum level (about 6 to 8-fold) after 20–30 min. Along with ClpP and HtrA proteases, Lon maintains protein quality control in *C. jejuni*, preventing excessive accumulation of misfolded proteins. In particular, a *C. jejuni* Δ*clpP*/Δ*lon* double mutant was impaired in its growth after puromycin-triggered protein misfolding [84]. In contrast, the corresponding single Δ*clpP* and Δ*lon* deletion mutants formed colonies similar to the wt *C. jejuni*, suggesting that both proteases eliminate misfolded proteins interchangeably. In line with the above observations, a Δ*clpP*/Δ*lon* double mutant, but not the single *C. jejuni* mutants, showed increased accumulation of misfolded proteins [84]. Finally, all Δ*clpP*/Δ*lon* double and single *C. jejuni* mutants exhibited reduced abilities to invade epithelial cells, indicating a major role of these quality control proteins in *C. jejuni* virulence, perhaps by processing precursors of several pathogenicity factors [84]. Overall, this chaperone and protease function appears to be crucial for the bacteria as our genome screens revealed presence of the *lon* gene in all *C. jejuni* genomes in the NCBI RefSeq Genome Database and in all 34 analyzed *Campylobacter* species (Table 1).

#### 2.2.3. FtsH (Cj1116c)

Similar to Lon, FtsH proteases possess an AAA+ module linked to the protease domain. The N-terminal transmembrane domain is followed by the large and small AAA+ domains that form a single AAA+ module. Finally, the C-terminus of the AAA+ module is flanked by the protease domain. The major roles of FtsH in bacteria include quality control and degradation of membrane proteins, heat shock response and regulation of lipopolysaccharide biosynthesis [91]. FtsH was also shown to be essential in maintaining bacterial virulence of *Edwardsiella piscicida* during infection of Zebrafish; FtsH deficiency resulted in reduced bacterial adhesion, internalization and intracellular survival [92]. While it is likely that *C. jejuni* FtsH is involved in maintenance of membrane proteins and LPS, similar to FtsH in many other bacteria and consistent with the predicted location at the cytoplasmic membrane (Table 1), the specific role of FtsH in *C. jejuni* lifestyle and pathogenesis still needs to be investigated. Unfortunately, attempts to create a *C. jejuni* Δ*ftsH* knockout mutant were so far unsuccessful [93]. This observation and the universal presence of FtsH homologs among *C. jejuni* isolates and in all analyzed *Campylobacter* species suggest an essential role of the protease for bacterial survival.

### 2.3. PqqE Protease (Cj0805)

*C. jejuni* protease PqqE belongs to the M16B family of zinc-dependent proteases. In eukaryotes, this protease is known as mitochondrial processing peptidase (MPP). Given the bacterial origin of the mitochondria, the cellular location of this peptidase points to an evolutionary bacterial origin of the enzyme. In most bacteria, including *E. coli*, this enzyme is located in the cytoplasm. The *Vibrio vulnificus* secreted insulin-degrading protease SidC, however, possesses an N-terminal signal peptide that is absent from PqqE homologs in other bacteria and was found secreted into the environment [94]. Mouse infection studies indicated an important role of SidC in *V. vulnificus* virulence. Compared to wt bacteria, a Δ*sidC* deletion mutant was severely impaired in its pathogenicity and barely colonized mice. In addition, SidC showed insulin-degrading activity, and the infection of diabetic mice resulted in higher bacterial numbers of both wt and Δ*sidC* bacteria compared to the infection of wt mice. Apparently, hyperglycemia favored bacterial survival, growth and proliferation in the infected host [94]. Similar to SidC, PqqE from *H. pylori* was identified as part of the secretome [95] and was found inside *H. pylori* OMVs [96]. PqqE from *H. pylori* was reported to compromise the integrity of the gastric epithelial layer by cleavage of the junctional adhesion molecule JAM-A during *H. pylori* interaction with the gastric epithelial layer [97]. However, these data were obtained by indirect experiments, warranting confirmation by additional data using a *pqqE* knockout mutant. Interestingly, unlike HtrA that cut the extracellular domain of E-cadherin, PqqE was proposed to cleave JAM-A at the cytoplasmic domain, and thus inside the host cells. While PqqE from *H. pylori* was secreted and released in OMVs, an analysis in PSORTb v3.0 [98] predicted a cytoplasmic location for PqqE from *C. jejuni*, suggesting that this protein is not located at the bacterial cell surface and is likely not being secreted. Thus, as expected from this analysis, PqqE was not present in *C. jejuni* OMVs [63]. In addition, infection experiments with *C. jejuni* and *C. coli* did not result in the disruption of JAM-A [97], suggesting different functions of *H. pylori* and *C. jejuni* PqqE proteases during pathogen-host interaction. In conclusion, the function of PqqE during *C. jejuni* pathogenesis remains to be unraveled. Unfortunately, the creation of a *pqqE* gene deletion mutant in *C. jejuni* may be challenging, because *H. pylori* *pqqE* (HP1012) is present in all isolates [99], and as mentioned above, attempts to create *H. pylori* Δ*pqqE* mutants have so far failed [97]. Furthermore, our genome screening revealed universal presence of *pqqE* among *C. jejuni* isolates, and PqqE homologs were found in all analyzed *Campylobacter* species (Table 1). Together, the data suggest that this bacterial protease might be essential, and attempts to generate a deletion mutant have been unsuccessful so far [97].

## 3. Peptidases

### 3.1. Proline Aminopeptidase PepP (Cj0653c)

Proline aminopeptidases are widely distributed from prokaryotes to eukaryotes. They play important roles in maintaining cell viability, virulence and other functions [100,101,102,103]. Aminopeptidase P from *C. jejuni* was first identified by two-dimensional gel electrophoresis that showed specific expression of the protein in a strain that robustly colonized the intestinal tract of chicken [76]. Recently, *C. jejuni* proline peptidase P (PepP) was identified as a potential virulence factor [103]. PepP was separated by SDS-PAGE as a ~70 kDa protein band, which was further defined as an M24 family metallopeptidase of 596 amino acids (aa) in size (locus WP_002854975.1). As described in the Pfam database, the corresponding sequence encoded two N-terminal creatinase/prolidase domains (creatinase N and creatinase N2) followed by a X-prolyl amino-peptidase (APP) domain, and a C-terminal M24 peptidase domain. Of those, the APP domain was shown to hydrolyze the N-terminal residue of a substrate at the Xaa-Pro site [104].

**Figure 1:**
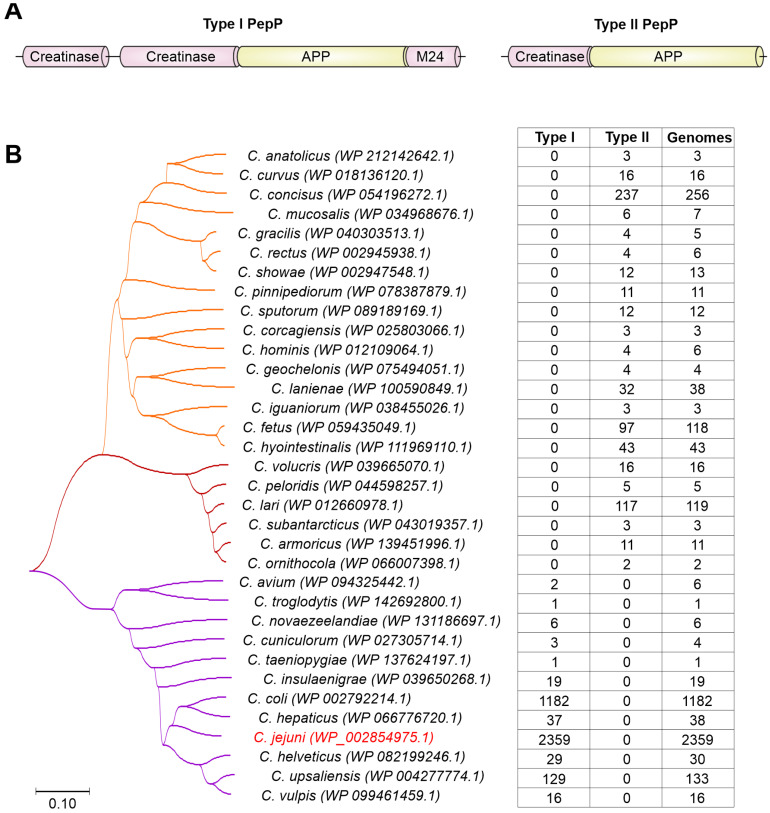
**Diversity of proline aminopeptidases found in *Campylobacter* spp.** (**A**) Proposed schematic domain organization of two PepP proteases indicated as Type I and Type II. One or two N-terminal Creatinase domains are followed by C-terminal X-Prolyl Aminopeptidase (APP) domain. Type I PepP additionally has a short M24 peptidase domain at the C-terminus. (**B**) Phylogenetic tree of PepP proteases found in *Campylobacter* spp. Type I and Type II PepP proteases are indicated by purple and brown/orange branches, respectively. Values in the table correspond to the number of genomes possessing either type I or type II PepP peptidases as identified by tblastn searches against the NCBI RefSeq Genome Database, and the total number of genomes per species in the database. All species have either type I PepP or type II PepP, but not both. In most species, all genomes possess the *pepP* gene.

The PepP peptidases are highly conserved among *C. jejuni* strains and other *Campylobacter* species, including *C. coli*, *C. hepaticus*, *C. upsaliensis* and others (Table 1). Interestingly, we have identified a shorter PepP variant in other *Campylobacter* species with a size of approximately 341 aa. Therefore, we propose to name the 596 aa long and 341 aa long PepP peptidases as type I and type II, respectively. The shorter PepP peptidase (type II) harbors an APP domain at the C-terminus, while the N-terminus likely encodes a creatinase domain (Figure 1A), as predicted by Conserved Domain Database [105]. Intriguingly, all *Campylobacter* species have only one type of PepP protease (either type I or type II), separating them into two distinct groups (Figure 1B), the type I group with the important human pathogens *C. jejuni* and *C. coli*, and the type II group with animal pathogens and commensals such as *C. fetus* and *C. lari*.

While the function of the type II PepP in *Campylobacter* spp. is currently still unclear, the type I PepP from *C. jejuni* was shown to contribute in murine campylobacteriosis [103]. In particular, a *C. jejuni* Δ*pepP* mutant caused less severe symptoms in microbiota-depleted IL10^−/−^ knockout mice and was associated with less pronounced apoptotic and innate immune cell (F4/80+) responses. Furthermore, intact PepP was required for an efficient *C. jejuni*-induced pro-inflammatory response in the intestine, including the release of interferon-γ (IFNγ), tumor necrosis factor (TNF), monocyte chemoattractant protein (MCP)-1 and IL-6. Although the *pepP* gene deletion did not compromise the colonization ability of *C. jejuni* in mice, it reduced the pro-inflammatory capacity of the bacterium. Interestingly, in other pathogens such as *Eikenella corrodens*, the prolyl aminopeptidase was reported to function as a hemolytic factor, significantly contributing to the pathogenicity of these bacteria [106]. Further research will shed more light on other potential activities of PepP in *C. jejuni* virulence.

### 3.2. Oligopeptidase PepF (Cj1099)

*C. jejuni* PepF is an oligopeptidase of the peptidase family M3B. PepF oligopeptidases are peptidases that commonly cleave short oligopeptides, but cannot degrade full-length proteins, hence the name. They have a broad substrate specificity and hydrolyse peptides between 5 and 21 amino acids in length [107,108]. Oligopeptide hydrolysis along with broad substrate specificity suggests that PepF peptidases are implemented in the degradation of cleavage products of various other proteases, and thus in protein recycling [109,110]. PepF oligopeptidases, including PepF from *C. jejuni*, contain a His-Glu-X-X-His motif that is followed by another glutamate residue, located 23 amino acids downstream. The two histidines and the downstream glutamate bind a single catalytic zinc ion and form the catalytic centre, while the glutamate residue embedded between the two histidines is believed to assist in the catalytic reaction by orienting the zinc ion and a water molecule [107]. 

In many bacteria, PepF peptidases are located in the cytoplasm. An analysis of the subcellular location using PSORT [98] confirmed a cytoplasmic localization for PepF from *C. jejuni* (Table 1). However, in *Bacillus amyloliquefaciens* PepF is secreted into the environment. Outside the bacterial cell, this peptidase apparently processes oligopeptides to generate pentapeptides, which represent small signal molecules that are subsequently imported into the cell and are involved in the initiation of sporulation [108]. While it is currently unknown whether *C. jejuni* PepF is involved in pathogen-host interactions and virulence, the presence of PepF peptidases in all *C. jejuni* isolates and in all examined *Campylobacter* species (Table 1) suggests conserved house-keeping functions such as peptide recycling or perhaps cell signaling.

### 3.3. C26 Peptidase (Cj1417c)

Another interesting peptidase that might play a major role in *C. jejuni* virulence is the C26 peptidase of the γ-glutamyl-gamma-aminobutyrate hydrolase family. Transposon mutagenesis coupled with sequencing approaches revealed significantly reduced *C. jejuni* colonization of mice upon inactivation of the C26 peptidase (CJJ81176_1416 in strain 81-176, Cj1417c in strain NCTC11168) [78]. The authors suggested that the peptidase may be involved in energy acquisition and/or protein biosynthesis that overall impacted the colonization capability by *C. jejuni*. These findings were consistent with a previous study that showed CJJ81176_1416 to be involved in chicken colonization by *C. jejuni* [111]. Furthermore, an earlier study showed that the acquisition of a gene encoding a γ-glutamyltranspeptidase enabled the bacteria to consume glutamine and glutathione, which enhanced their ability to colonize the intestine [11]. Interestingly, the observed growth or colonization effect may be strain-specific, because not all *C. jejuni* genomes possess the *cj1417c* gene. Our genome screen showed *cj1417c* presence in 1995 out of the 2359 genomes in the NCBI RefSeq Genome Database. In addition, 20 of the 34 analyzed *Campylobacter* species lack a Cj1417 protease homolog (Table 1). Thus, further studies focusing on the metabolic plasticity provided by peptidases may help to better understand the *C. jejuni*-driven pathogenesis. 

## 4. Evolution of Proteases and Peptidases in the Campylobacter Genus

**Figure 2:**
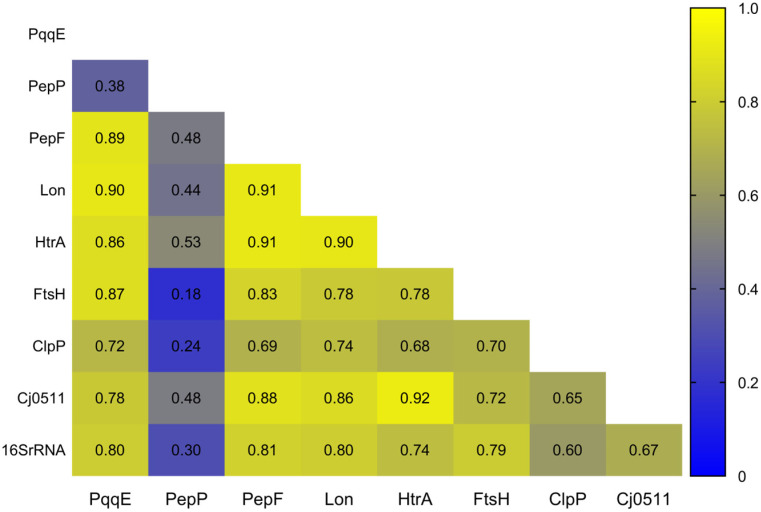
**Pairwise Pearson correlation coefficients r of similarity matrices from seven proteases/peptidases and the *16S rRNA* gene from 34 *Campylobacter* species**. High pairwise r values (yellow boxes) indicate congruent evolutionary history, low r values (blue boxes) indicate independent and distinct evolutionary trajectories. For each protease/peptidase and the *16S rRNA* gene, pairwise similarity matrices were calculated for all 34 *Campylobacter* species in MEGA X [112]. Protein analyses were conducted using the Poisson correction model, with ambiguous pairwise positions removed (pairwise deletion option). The *16S rRNA* gene matrix was calculated using the Maximum Composite Likelihood model with the pairwise deletion option to remove ambiguous positions. The pairwise Pearson correlation coefficients r of the eight resulting similarity matrices were calculated using the PEARSON function implemented in Microsoft Excel. The heatmap was generated in GraphPad Prism 9.

Seven of the 10 discussed proteases, i.e., HtrA, Cj0511, ClpP, Lon, FtsH, PepF, and PqqE, were found to be present in all 34 analyzed *Campylobacter* species (Table 1), suggesting that these proteases were likely present in the ancestor of the genus. In fact, these seven proteases were also found present in other ε-Proteobacteria, including *H. pylori*, *Helicobacter hepaticus*, *Wolinella suchinogenes* and even in the environmental ε-Proteobacteria *Sulfurospirillum arsenophilum* and *Nautilia profundicola*, indicating a common evolutionary origin and implying that these proteases likely have important house-keeping functions in the bacterial cell. Some of those enzymes (HtrA, Cj0511, PqqE) acquired additional functions in the interaction with animal/human hosts. As a result of the common origin and subsequent evolution and diversification along with the other genes in the genomes, the pairwise similarity matrices of these genes were quite similar to each other and displayed high pairwise Pearson correlation coefficients ranging from r = 0.65 to r = 0.91 (Figure 2). In addition, the evolution was congruent with that of the *16S rRNA* gene with scores of r = 0.60 to r = 0.81. In contrast, PepP showed low Pearson coefficients ranging from r = 0.18 to r = 0.53, indicating a different evolutionary trajectory.

**Figure 3:**
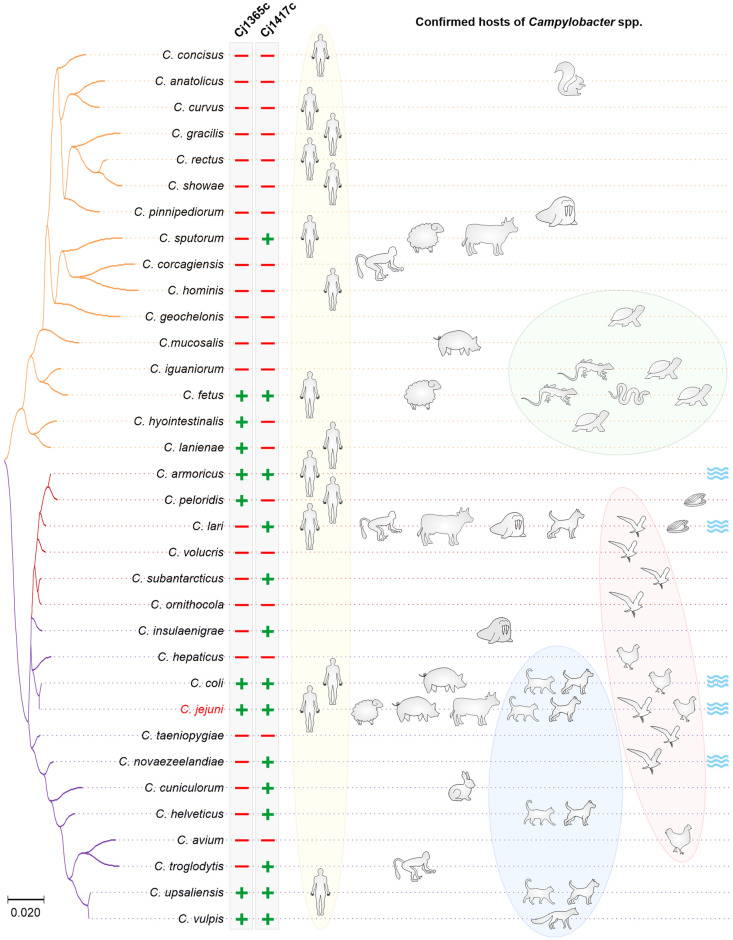
**16S rRNA-based Neighbor-joining tree of 34 Campylobacter species, and corresponding hosts.** The color-coded tree branches indicate type I (purple) and type II (orange and brown) PepP variants as in Figure 1. Presence or absence of Cj1365c or Cj1417c (C26) proteases in the Campylobacter species is indicated by “plus” and “minus” symbols, respectively. Confirmed human [113,114,115,116,117,118,119,120,121,122,123,124,125,126,127,128], monkey [115,129,130], sheep [117,120], pig [117,131,132,133,134], ruminant [115,117,120,135], marine mammal [115,136,137], poultry [113,117,138,139], cat/dog [113,115,118,140,141], fox [142], rabbit [143], squirrel [144], wild bird [115,145,146,147,148,149,150], reptile [151,152,153,154], and sea shell [116] hosts of Campylobacter spp., as well as water source [113,115,119,148], are shown on the right side by the corresponding symbols. Yellow, blue, red and green ellipses show the “human”, “cat/dog/fox”, “bird” and “reptile” host groups, respectively.

Indeed, as mentioned above, protease PepP was present as two very distinct protein versions. Type I contained four protein domains (creatinase N, creatinase N2, APP, M24). The much shorter type II contained only a creatinase N domain (with weak homology) and an APP domain. In addition, the shorter type II split into two groups in the phylogenetic tree (Figure 1). Interestingly, superimposing the PepP protein variants onto the *16S rRNA*-based phylogenetic tree revealed specific clustering of the PepP variants (Figure 3). Species carrying the short type II peptidase (orange tree branch, as in Figure 1) clustered together in a distinct branch of the phylogenetic tree. However, the other major branch of the *16S rRNA* gene-based tree contained two groups of species possessing either type I or type II PepP peptidase variants (purple and brown tree branches, respectively). An analysis of the chromosomal location of *pepP* revealed that all type I *pepP* gene homologs are located at gene position *cj0653c*. In contrast, all type II *pepP* homologs from both orange and brown clades are located between *cj0065c* and *cj0066c* gene homologs. Given that the orange and brown species clades are located at different branches of the *16S rRNA*-based phylogenetic tree, this suggests type II PepP to be ancestral in *Campylobacter*.

In addition, type II PepP homologs are also present in multiple other species of the ε-Proteobacteria, including the closely related genera *Helicobacter*, *Wolinella* and *Sulfurospirillum*, all of which belong to the order *Campylobacterales*, and the termophilic *Nautilia* of the order *Nautiliales*, supporting the proposed type II *pepP* gene ancestry in *Campylobacter*. In contrast, type I PepP peptidase homologs were only found in more distantly related genera, including *Neisseria*, *Lautropia* (both β-Proteobacteria), *Acinetobacter* and *Pseudomonas* (both γ-Proteobacteria), suggesting a secondary gene import from a currently unknown source into the ancestor of the purple species clade.

The C26 peptidase (Cj1417c) was only present in a subset of 14 *Campylobacter* species (Table 1). Notably, we found C26 peptidase homologs in *W. succinogenes* and *H. hepaticus*, but not in *H. pylori* and also not in the environmental *Nautilia* and *Sulfurospirillum* bacteria (Table 1). All 14 *Campylobacter* species harbored gene *cj1417c* at the same chromosomal location, suggesting a joint evolutionary origin of this peptidase in the genus. We found Cj1417c present in multiple species of the brown and purple clades, indicating loss of the *cj1417c* gene in individual species and lineages (Figure 3). In contrast, only two species (*C. fetus*, *C. sputorum*) in the orange clade possessed Cj1417c. Therefore, multiple species of the orange clade must have lost the gene. Alternatively, *cj1417c* may have been lost in the ancestor of the entire clade, followed by independent acquisition in the two species. However, the latter scenario is less likely, because the pairwise genetic distances between the 14 different Cj1417c homologs strongly support a common evolutionary origin and subsequent gene loss.

The evolutionary history of Cj1365c in *Campylobacter* appears to be different. First, this protease is only present in nine of the 34 analyzed species (Table 1, Figure 3). However, whether protease Cj1365c is present in all isolates of these nine species remains to be analyzed. Second, other ε-Proteobacteria such as the already mentioned *W. succinogenes*, *H. hepaticus*, *H. pylori*, *N. profundicola* and *S. arsenophilum* lack this protease (Table 1), which argues against a common evolutionary origin. Third, as outlined above, we found that not all *C. jenuni* and *C. coli* isolates possess this protease; we identified the *cj1365c* gene in 1845 of 2359 *C. jejuni* genomes in the RefSeq Genome Database. In addition, only a small subset of the *C. coli* genomes possess this gene, likely because of the recent gene import from *C. jejuni* into the same chromosomal location. While in *C. jejuni* and *C. coli* the gene is located at position *cj1365c*, it is located between *cj0067* and *cj0068* gene homologs in *C. armoricus* and *C. peloridis*, between *cj1368* and *cj1369* gene homologs in *C. upsaliensis* and *C. vulpis*, between the gene homologs of *cj0027* and *cj1615* in *C. hyointestinalis* and *C. lanienae* and inserted between two copies of the *cj1153* gene homolog in *C. fetus*. These multiple chromosomal locations indicate that these species likely acquired the gene independently from (a) currently unknown source(s).

## 5. Conclusions and Perspectives

*C. jejuni* is the major food-borne pathogen of the human intestine. Infection is mainly caused by the consumption of contaminated poultry meat products or raw milk. The pathogen induces campylobacteriosis and—in rare, but relevant cases—severe post-infectious diseases as discussed above. These *C. jejuni*-associated sequelae result in enormous socioeconomic costs of billions of dollars yearly worldwide. However, the molecular mechanisms and bacterial factors implicated in disease development are not entirely understood. Here we identified and discussed the key functions of proteases in *C. jejuni* and related species. Proteases represent widespread enzymes with major physiological functions, and proteases also serve as efficient weapons during infection, as utilized by various other bacterial pathogens [21,22,23]. In case of *C. jejuni*, it appears that we are just beginning to unravel the multitude of proteases and their specific virulence properties. The only host cell substrates identified so far are the cell-to-cell junctional proteins occludin, claudin-8 and E-cadherin that are cleaved by serine proteases HtrA with verified cleavage sites [14]. Other data point to cleavage of junction proteins and of the endoplasmic reticulum chaperone protein BiP/GRP78 by Cj1365c [45], but these observations need to be confirmed by in vivo infection experiments. The potential host cell substrates and preferred cleavage sites of any other of the above discussed *C. jejuni* proteases are still unknown. Thus, more work needs to done to unravel the function of all these proteases in pathogen-host interactions. In addition, the catabolism of *C. jejuni* in the colon is highly specific, including the acquisition of amino acids such as aspartate and asparagine as mentioned above [25]. However, whether secreted *C. jejuni* proteases play a role in the acquisition of these amino acids, such as through degradation of extracellular proteins, is currently unknown and constitutes a topic for future studies. It may be speculative whether the presence of multiple proteases might help *Campylobacter* species to colonize/infect a variety of different hosts. However, there appears to be a tendency that *Campylobacter* species possessing both Cj1365c and Cj1417c (e.g., *C. jejuni*, *C. coli*, *C. upsaliensis*, *C. fetus*) are capable of colonizing multiple hosts, including hosts as different as mammals and birds or hosts as different as mammals and reptiles (Figure 3). Yet, there are exceptions; some species that possess both proteases are currently known to colonize only a single particular host, such as *C. vulpis* or *C. armoricus*. In addition, we need to keep in mind that not all *Campylobacter* species colonize the lower digestive tract, some were isolated from the oral cavity. In general, our knowledge of bacterial proteases and their importance in pathogenic processes is still limited, including whether proteases play a role in the development of specific diseases such as RA, GBS, MFS or IBD. In addition, the gut microbiota constitutes a complex microenvironment that affects human health and disease, and we are only beginning to unravel the complex interactions between host, microbiota and pathogens. Here, secreted bacterial proteases are of particular interest and constitute a special class of enzymes used by many bacteria to shape their surrounding ecosystem. Regarding the variety of activities and known targets of these proteases, only HtrA has so far been investigated for its potential as therapeutics against *C. jejuni*. Further research on HtrA and other *C. jejuni* proteases is an important task for the near future. Here, the array of *C. jejuni* proteases and their target molecules represent an increasingly important research topic area in the human health sector.

## Figures and Tables

**Table 1:** Distribution of various proteases within the *Campylobacter* genus and in other ε-Proteobacteria.

Protease Locus Cj	HtrA 1228c	0511	1365c	ClpP 0192c	Lon 1073c	FtsH 1116c	PepP 0653c	PepF 1099	PqqE 0805	C26 1417c
Predicted cellular location	P	CM, P	OM, EC	C	C	CM	C	C	C, CM, P	C
*C. jejuni*	Y	Y	Y	Y	Y	Y	Y	Y	Y	Y
*C. armoricus*	Y	Y	Y	Y	Y	Y	Y	Y	Y	Y
*C. vulpis*	Y	Y	Y	Y	Y	Y	Y	Y	Y	Y
*C. fetus*	Y	Y	Y	Y	Y	Y	Y	Y	Y	Y
*C. upsaliensis*	Y	Y	Y	Y	Y	Y	Y	Y	Y	Y
*C. coli*	Y	Y	Y	Y	Y	Y	Y	Y	Y	Y
*C. troglodytis*	Y	Y	-	Y	Y	Y	Y	Y	Y	Y
*C. lari*	Y	Y	-	Y	Y	Y	Y	Y	Y	Y
*C. cuniculorum*	Y	Y	-	Y	Y	Y	Y	Y	Y	Y
*C. helveticus*	Y	Y	-	Y	Y	Y	Y	Y	Y	Y
*C. insulaenigrae*	Y	Y	-	Y	Y	Y	Y	Y	Y	Y
*C. subantarcticus*	Y	Y	-	Y	Y	Y	Y	Y	Y	Y
*C. sputorum*	Y	Y	-	Y	Y	Y	Y	Y	Y	Y
*C. novaezeelandiae*	Y	Y	-	Y	Y	Y	Y	Y	Y	Y
*C. peloridis*	Y	Y	Y	Y	Y	Y	Y	Y	Y	-
*C. lanienae*	Y	Y	Y	Y	Y	Y	Y	Y	Y	-
*C. hyointestinalis*	Y	Y	Y	Y	Y	Y	Y	Y	Y	-
*C. volucris*	Y	Y	-	Y	Y	Y	Y	Y	Y	-
*C. concisus*	Y	Y	-	Y	Y	Y	Y	Y	Y	-
*C. corcagiensis*	Y	Y	-	Y	Y	Y	Y	Y	Y	-
*C. curvus*	Y	Y	-	Y	Y	Y	Y	Y	Y	-
*C. gracilis*	Y	Y	-	Y	Y	Y	Y	Y	Y	-
*C. pinnipediorum*	Y	Y	-	Y	Y	Y	Y	Y	Y	-
*C. rectus*	Y	Y	-	Y	Y	Y	Y	Y	Y	-
*C. showae*	Y	Y	-	Y	Y	Y	Y	Y	Y	-
*C. mucosalis*	Y	Y	-	Y	Y	Y	Y	Y	Y	-
*C. avium*	Y	Y	-	Y	Y	Y	Y	Y	Y	-
*C. geochelonis*	Y	Y	-	Y	Y	Y	Y	Y	Y	-
*C. hepaticus*	Y	Y	-	Y	Y	Y	Y	Y	Y	-
*C. hominis*	Y	Y	-	Y	Y	Y	Y	Y	Y	-
*C. iguaniorum*	Y	Y	-	Y	Y	Y	Y	Y	Y	-
*C. taeniopygiae*	Y	Y	-	Y	Y	Y	Y	Y	Y	-
*C. anatolicus*	Y	Y	-	Y	Y	Y	Y	Y	Y	-
*C. ornithocola*	Y	Y	-	Y	Y	Y	Y	Y	Y	-
*H. pylori*	Y	Y	-	Y	Y	Y	Y	Y	Y	-
*H. hepaticus*	Y	Y	-	Y	Y	Y	Y	Y	Y	Y
*W. succinogenes*	Y	Y	-	Y	Y	Y	Y	Y	Y	Y
*S. arsenophilum*	Y	Y	-	Y	Y	Y	Y	Y	Y	-
*N. profundicola*	Y	Y	-	Y	Y	Y	Y	Y	Y	-

Y present; - absent; Cellular location as predicted by Motif Search (https://www.genome.jp/tools/motif/ accessed on 23 January 2023) and/or PSORTb v3.0: P periplasmic; CM cytoplasmic membrane; OM Outer membrane; EC extracellular; C cytoplasmic. Other ε-Proteobacteria: *Helicobacter pylori*, *Helicobacter hepaticus*, *Wolinella succinogenes*, *Sulfurospirillum arsenophilum*, *Nautilia profundicola*.

## Data Availability

Not applicable.
